# Supplementary material for: Simple scoring tool to estimate risk of hospitalization and mortality in ambulatory and emergency department patients with COVID-19
Source: PLoS One. 2022 Mar 3;17(3):e0261508. doi: 10.1371/journal.pone.0261508 (PMC8893609; doi:10.1371/journal.pone.0261508)
Supplement: Attachment — Submitted filename: Response to reviewers.docx [file pone.0261508.s002.docx]

November 11, 2021

Re: PONE-D-21-06171

Dear Dr. Torti,

Thank you for the thorough peer review or our original research article entitled “Simple Scoring Tool to Estimate Risk of Hospitalization and Mortality in Ambulatory and Emergency Department Patients with COVID-19” (PONE-D-21-06171).

We have responded to the excellent suggestions by the reviewers. Please point-by-point table below. In response to comments by reviewers and editorial staff, have now conducted a robust independent validation in a very large, temporally-distinct cohort including more than 80,000 patients. This secondary validation cohort spans a different time period and addresses possible influence by changes in patient characteristics or the Delta variant. We have also included comparative validation of the Charlson Comorbidity index in our dataset and contrast our model with other published models in the discussion.

We believe the paper is now significantly improved and propose that the current work be considered for publication with this validation basis.

We have submitted a deidentified dataset with this revision.

Please see below for a revised conflicts of interest statement and author contributions.

Thank you for your consideration of this manuscript.

Sincerely,

Brandon J. Webb, MD, Corresponding author

Nicholas M. Levin, MD, Alternate Corresponding author

Brandon.Webb@imail.org

Nicholas.Levin@hsc.utah.edu

Conflicts of Interest: IP reports salary support through a grant from the National Institutes of Health (U.S.A). SB reports salary support from the U.S. NIH, Centers for Disease Control and the Department of Defense; he also reports receiving support for chairing a data and safety monitoring board for a respiratory failure trial sponsored by Hamilton, effort paid to Intermountain for steering committee work for Faron Pharmaceuticals and Sedana Pharmaceuticals for ARDS work, support from Janssen for Influenza research, and royalties for books on religion and ethics from Oxford University Press/Brigham Young University. BW reports partial salary support from a U.S. Federal grant from AHRQ. ES receives partial salary support through grants from the Centers for Disease Control. At the time of submission, Intermountain Healthcare and the University of Utah have participated in COVID-19 trials sponsored by: Abbvie, Genentech, Gilead, Regeneron, Roche, and the U.S. National Institutes of Health ACTIV and PETAL clinical trials networks; several authors (BW, IP, JB, SB, ES) were site investigators on these trials but received no direct or indirect remuneration for their effort. ES, BJW, SMB and MS are members of the Utah crisis standards of care scarce medication committee. This does not alter our adherence to PLOS ONE policies on sharing data and materials.

Author Contributions:

Study concept: BJW, JB, IP, PJ, DH, BH, AS, NS, WB, EH, DM, RS, SMB

Study design: BJW, JB IP, SMB, GS

Data collection: BJW, NG

Statistical analysis: BJW, JB, IP, SMB

Interpretation of results: All authors

Manuscript preparation: All authors

Critical review of the manuscript: All authors

| **Reviewer Comments** | **Author Response** |
| --- | --- |
| **Reviewer #1:** The authors propose a new model capable of stratifying the risk of hospitalization and mortality in patients with COVID-19 outpatient or who access the emergency department.  The strengths of the score are:  1) the sample size and, consequently, the number of the score validation population;  2) the simplicity of the score, as it uses anamnestic and non-laboratory data.  Nonetheless, several issues need to be addressed by the authors: | Thank you for the comments. |
| 1) Like all scores, it requires external validation, as the data is drawn from what is observed. For example, a state's health system, whether public or private, certainly influences health care towards the weakest and poorest social classes. This data can have repercussions on the results of the score. It is no coincidence that belonging to a more impoverished community represents a risk factor (OR 1.8, CI95% 1.5-2.2, p-value <0.0001). | We agree. To further enhance validation, we have now conducted a second, independent validation in a temporally distinct cohort of more than 80,000 patients. |
| 2) A second observation regards the category of immunosuppressed patients. First of all, this label includes a multitude of different patients (recipient of a solid organ or hematopoietic stem cell transplant, on chemotherapy, biologic or other immunosuppressive agents targeting B or T cell activity, chronic corticosteroids at a prednisone-equivalent dose of 20 mg per day or more significant for more than 30 days, human immunodeficiency virus complicated by acquired immunodeficiency syndrome (AIDS), heritable immunodeficiency). It is hard to think that an HIV patient is similar to an immunodeficient patient because transplanted. Also, because the data in the literature show that some immunosuppressive states could even be protective. Furthermore, terminal metastatic patients not eligible for treatment have been excluded, and, consequently, we have no data on this population, which also falls into the category of patients considered "fragile". Being 6 months old doesn't make COVID infection any less dramatic. So, I would suggest breaking the "immunosuppression" label into its components and analyzed individually. | Thank you for the excellent comment. We agree that not all patients with immunocompromised conditions have equivalent risk for hospitalization. However, because the numbers of immunocompromised patients in our cohort were quite low, we are unable to break them in to more granular categories without having too few events to be retained in the regression model. |
| 3) Finally, I have an objection to the usefulness of introducing a new score without making a comparison with those already present. For example, it would be nice to compare the data obtained against a known and validated score like the Charlson comorbidity index, which is similar to that proposed. | Excellent suggestion. We conducted a validation of Charlson comorbidity index in our dataset. Results of this analysis have been added to the manuscript. |
| **Reviewer #2** |  |
| Methodology: Appropriate and informative. IRB Approval/ Number is needed. | We have added the IRB approval information to the manuscript. |
| Results: Calibration of the final multivariate logistic regression model (The Hosmer–Lemeshow test) is needed.  The Discrimination of the final multivariate logistic regression model should be assessed by the area under the receiver operator characteristic (ROC) curve. | We intentionally did not use Hosmer Lemeshow because the size of our cohort is so large that the H-L goodness of fit test is no longer valid. We have included references supporting this choice. Instead, we report the fit of the model using the R^2^ metric. We do report the AUROC for the model. |
| Discussion: Appropriate, however I expected more comparisons with recent studies on the same topic. | Thank you for the suggestion. We have added a paragraph to the discussion that sets our study against the context of other papers describing predictive models. |
| References: Please use Vancouver’s Style in all references. | This has been changed to Vancouver |
| Additional Editor Comments:  Unfortunately, your work lacks external validation and comparison with known and validated risk scores. For these reasons, it is not suitable for publication as it appears now. | Thank you. We have now included a very large and robust independent validation cohort with more than 80,000 patients, which spans a different time period that also reflects possible influence by changes in patient characteristics or the Delta variant. We have also included comparative validation of the Charlson Comorbidity index in our dataset and contrast our model with other published models in the discussion. |
